# The white matter characteristic of the genu of corpus callosum coupled with pain intensity and negative emotion scores in patients with trigeminal neuralgia: a multivariate analysis

**DOI:** 10.3389/fnins.2024.1381085

**Published:** 2024-03-21

**Authors:** Baijintao Sun, Chuan Zhang, Kai Huang, Anup Bhetuwal, Xuezhao Yang, Chuan Jing, Hongjian Li, Hongyu Lu, Qingwei Zhang, Hanfeng Yang

**Affiliations:** ^1^Department of Radiology, Affiliated Hospital of North Sichuan Medical College, Nanchong, China; ^2^Center for Brain Imaging, School of Life Science and Technology, Xidian University, Xi’an, Shaanxi, China

**Keywords:** trigeminal neuralgia, negative emotion, partial least squares, structural magnetic resonance imaging, pain

## Abstract

**Background:**

Trigeminal neuralgia (TN) is a chronic neuropathic pain disorder that not only causes intense pain but also affects the psychological health of patients. Since TN pain intensity and negative emotion may be grounded in our own pain experiences, they exhibit huge inter-individual differences. This study investigates the effect of inter-individual differences in pain intensity and negative emotion on brain structure in patients with TN and the possible pathophysiology mechanism underlying this disease.

**Methods:**

T1 weighted magnetic resonance imaging and diffusion tensor imaging scans were obtained in 46 patients with TN and 35 healthy controls. All patients with TN underwent pain-related and emotion-related questionnaires. Voxel-based morphometry and regional white matter diffusion property analysis were used to investigate whole brain grey and white matter quantitatively. Innovatively employing partial least squares correlation analysis to explore the relationship among pain intensity, negative emotion and brain microstructure in patients with TN.

**Results:**

Significant difference in white matter integrity were identified in patients with TN compared to the healthy controls group; The most correlation brain region in the partial least squares correlation analysis was the genus of the corpus callosum, which was negatively associated with both pain intensity and negative emotion.

**Conclusion:**

The genu of corpus callosum plays an important role in the cognition of pain perception, the generation and conduction of negative emotions in patients with TN. These findings may deepen our understanding of the pathophysiology of TN.

## 1 Introduction

Trigeminal neuralgia (TN) is characterized by sudden stabbing, sharp, or electric shock-like sensations in regions dominated by the trigeminal nerve (Dou et al., 2016). Additionally, some patients also experience continuous pain concurrently, often accompanied by a propensity toward anxiety and depression (Dou et al., 2016; Bick and Eskandar, 2017; Zakrzewska et al., 2017; Hu and Iannetti, 2019). The etiology of trigeminal neuralgia has long been attributed to the aberrant neurovascular system (Bick and Eskandar, 2017), increased nociceptive afferent input to the brain, and structurally maladaptive changes. For clinical purposes, understanding the individual differences in brain abnormalities among patients with TN is crucial. Neuroimaging studies have revealed reproducible findings of abnormal brain structural changes (Zhang et al., 2020), while the core areas of the brain associated with patients’ pain symptoms remain largely unclear.

Insights from structural magnetic resonance imaging (sMRI) have suggested that reduced gray matter volume in the left inferior temporal gyrus (Wang et al., 2017) and the right precentral gruy (Ge et al., 2023) is negatively associated with pain intensity. These studies highlight changes in brain gray matter volume that may be linked to inter-individual symptoms. Diffusion tensor imaging (DTI) studies reveal that TN patients with lower white matter properties in the left anterior corona radiata, left external capsule, left cerebral peduncle (Liu et al., 2018) and genu of corpus callosum (Li et al., 2021a) exhibit abnormally higher pain intensity. These findings indicate that the integrity of white matter properties may also serve as a neuroimaging feature for individual differences in pain symptoms in TN patients. TN-affected patients commonly experience a high prevalence of negative emotions, including anxiety and depression (Talbot et al., 2019; Gilam et al., 2020). Two studies aimed to evaluate improvements in anxiety and depression in TN patients after operative responses, and they found significant improvements in anxiety and depression when TN pain was alleviated after surgical treatment (Cheng et al., 2017; Fan et al., 2021). These studies have demonstrated a strong correlation between variations in pain symptoms and individuals’ emotional states. However, associations between abnormal brain structure, pain intensity, and negative emotion were predominantly analyzed through mass univariate analysis. Univariate analysis assumes independence among all voxels and is not hierarchy, a hypothesis that cannot be true in the brain and is thus limited by the challenge of multiple comparisons.

In the current study, we hypothesized that the brain structure of TN patients would undergo gradual changes with increasing pain intensity and negative emotion, and that these altered regions may exhibit varying degrees of prominence (i.e., the contribution of the brain in one region are greater than in another). To test our hypothesis, we utilized sMRI and DTI to estimate whole-brain gray matter and white matter properties, respectively. Pain-related and emotion-related questionnaires were employed to assess the patient’s composite symptoms experienced by TN patients. Finally, we applied partial least squares (PLS) correlation analysis to assess the relationship among abnormal brain matter, pain intensity, and negative emotion in patients with TN.

## 2 Materials and methods

### 2.1 Ethics and patient consents

Our research adhered to the principles outlined in the Declaration of Helsinki, and approval for the study was granted by the Ethics Board of the Affiliated Hospital of North Sichuan Medical College. Participants provided written informed consent, which was voluntarily signed before the commencement of the experimental procedures. The MR image data were anonymized to ensure confidentiality during subsequent analysis.

### 2.2 Participants

We enrolled 46 patients (31 females and 15 males, average age 57.7 years) who were diagnosed with primary TN between December 2022 and June 2023 at the Affiliated Hospital of North Sichuan Medical College. Additionally, we recruited 35 healthy controls (18 females and 17 males, with an average age of 54.5 years) in our hospital. The exclusion criteria for healthy controls were the same as those for the TN group.

The inclusion criteria for the patients with TN were: (1) meeting the International Classification of Headache Disorders criteria (third edition) [Headache Classification Committee of the International Headache Society (IHS), 2018]; (2) disease duration longer than 3 months without surgical treatment; (3) right-handed individuals aged between 40 and 80 years; and (4) discontinuation of disease-related drugs (e.g., carbamazepine tablets) for more than 24 h.

The inclusion criteria for the healthy controls were: (1) right-handed individuals aged between 40 and 80 years; (2) discontinuation of vasoactive drug last three months.

Exclusion criteria for the patients with TN were: (1) presence of other mental or neurological illnesses (such as epilepsy or dementia, etc.); (2) experiencing other types of chronic pain (e.g., dysmenorrhea, migraine, or chronic headache, etc.); (3) history of alcohol or illicit drug abuse; (4) MRI contraindications, including claustrophobia and metallic implants or devices in the body.

### 2.3 Clinical measurements and imaging acquisition

The patients’ basic clinical information, including gender, age, affected side, disease duration, and relevant medication history, was collected. The healthy controls (HC) group also provided basic clinical information, including gender and age. All TN patients completed pain-related and emotion-related questionnaires, including the McGill Pain Questionnaire (MPQ) (Melzack, 1975), the Penn Facial Pain Scale (FPS) (Symonds et al., 2018), the Pain Anxiety Symptom Scale (PASS) (Rogers et al., 2020), and the Pain Catastrophic Scale (PCS) (Darnall et al., 2017). The MPQ consists of three parts, with scores ranging from 0 to 21, to assess patients’ pain intensity and emotion. The FPS is a 16-item survey with scores ranging from 0 to 100, evaluating patients’ pain symptoms in the past week and the current week. The PASS is a 20-item survey, and the PCS is a 13-item survey, both assessing negative emotion in the current situation.

Participants with TN underwent MRI scanning on a Siemens Skyra 3.0 T scanner with a 32-channel head coil in our hospital. The subjects were secured to the head with a foam sponge and instructed to remain still, relaxed, close their eyes, stay awake, and breathe quietly during the scan. The scanning range includes the entire cerebral hemisphere and cerebellum. DTI images were acquired using an echo-planer imaging sequence, and the parameters are as follows: repetition time (TR) = 8,500 ms; echo time (TE) = 92 ms; NEX = 1; matrix resolution = 128 × 128; field of view (FOV) = 240 mm × 240 mm; slices = 45; slice thickness = 3 mm without gap; and 30 nonlinear dispersion gradients (b = 1,000 s/mm^2^). 3D T1-weighted image acquisition parameters are as follows: TR = 2,300 ms; TE = 3 ms; TI = 900 ms; slice thickness = 1 mm without gap; FOV = 240 mm × 240 mm; matrix resolution = 256 × 256; flip angle = 9°; and NEX = 1.

### 2.4 Magnetic resonance imaging data processing and analysis

#### 2.4.1 Voxel-based morphometry analysis

We conducted whole-brain voxel-based morphometry (VBM) analysis with Statistical Parametric Mapping 12 software (www.fil.ion.ucl.ac.uk/spm). This technique facilitates voxel-wise statistical analysis by employing fully automated segmented gray matter, white matter, and cerebral spinal fluid (CSF). Following this, the affine registration algorithm was utilized to map all native-space tissue segments to the standard Montreal Neurological Institute (MNI) templates (voxel size: 1 × 1 × 1 mm^3^). Difeomorphic Anatomical Registration Through Exponentiated Lie Algebra (DARTEL) was employed for refining inter-subject registration through difeomorphic anatomical registration. The normalized images underwent smoothing with an isotropic Gaussian kernel of 8 mm full-width at half-maximum (FWHM) to facilitate statistical analysis. Whole-brain gray matter volume, white matter volume, and CSF volume were calculated and averaged for each group.

#### 2.4.2 Regional white matter diffusion property analysis

The DTI data underwent standard pre-processing steps using FMRIB Software Library (FSL) software.^1^ The DTIFIT function was applied to reconstruct diffusion tensors for each voxel through linear regression, obtaining fractional anisotropy (FA) values. FA represents the degree of tissue anisotropy and indicates the integrity of the white matter. Pre-processing steps included the correction of diffusion data for eddy currents and head movement artifacts. Subsequently, the Brain Extraction Toolbox (BET) was employed to extract brain masks from brain tissues, and FA images were generated by scaling the diffusion tensor to the DTI data using FMRIB’s diffusion toolbox (FDT 4.0).

**Figure 1:**
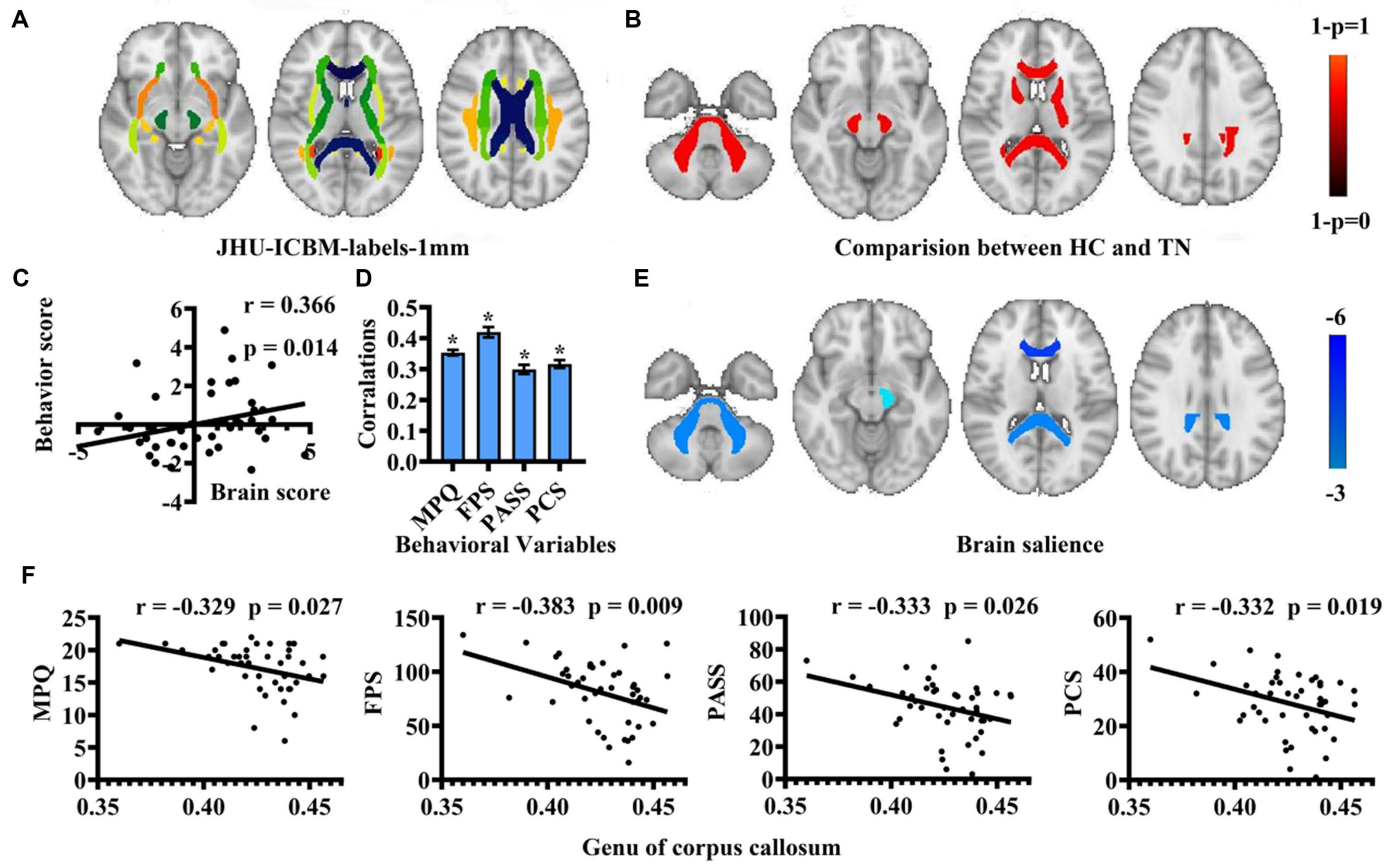
Regional white matter diffusion property and PLS correlation analysis results. **(A)** Utilization of JHU-ICBM-labels in analysis. **(B)** Identification of abnormal white matter regions in patients with TN compared to HCs depicted in warm colors. **(C)** Correlation between behavioral scores and brain scores of LV1 presented through a scatter plot. **(D)** A bar graph illustrates Pearson’s correlation coefficients between behavioral variables and brain scores of LV 1 for TN. (* means that the behavioral variable has correlated with brain scores). **(E)** Brain structures exhibiting associations with pain intensity and negative emotion are highlighted in cold colors. **(F)** Scatter plot showcasing the correlation among behavioral scores with the genu of the corpus callosum. (HC, healthy controls; TN, trigeminal neuralgia; MPQ, McGill Pain Questionnaire; FPS, Penn Faces Pain Scale; PASS, Pain Anxiety Symptom Scale; PCS, Pain Catastrophic Scale).

Regional white matter diffusion properties were analyzed to explore variations in FA values between patients with TN and HCs using FSL. Specifically, FA images for each subject were nonlinearly registered to a template of the averaged FMRIB58-FA standard space^2^ and aligned to the MNI space. The mean value of FA from each region in the JHU-White Matter-labels was calculated (Figure 1A).

### 2.5 Statistical analysis

We conducted two-sample t-tests for age and chi-square tests for gender to examine individual differences. In the VBM study, group comparisons between patients with TN and the HCs were carried out to identify areas of increased and decreased abnormality, and these were analyzed using t-tests. Age and gender were included as covariates in this analysis. The results were corrected for multiple comparisons using family-wise error (FWE), and were thresholded at *p* < 0.05 at the cluster level.

For white matter analysis, we investigated differences in FA value between patients with TN and the HCs using t-tests. Permutation tests with 5,000 iterations were employed for group-wise statistics. The results were corrected for multiple comparisons using FWE, and were thresholded at *p* < 0.05 at the cluster level.

### 2.6 Multivariate PLS correlation analysis

Multivariate PLS correlation analysis was performed using the graphical user interface from the Rotman Research Institute, Baycrest Centre, University of Toronto (downloaded from^3^ MATLAB). This analysis aimed to explore co-variations between the clinical behavioral variables (MPQ, FPS, PASS, and PCS) and abnormal white matter areas in patients with TN. We can obtain some of latent variables (LVs) with corresponding singular values [representing the proportion of the covariance accounted for by the given LV (the cross-block covariance)] and saliences (indicating the degree to which each variable was related to the effect characterized by that LV). *p* < 0.05 were considered statistically significant. The 5,000 bootstrap samples were constructed to evaluate the reliability of the effect on each voxel. Brain and behavior saliences were recalculated for each bootstrap sample. *p* < 0.01 were considered statistically significant.

## 3 Results

### 3.1 Demographics and clinical measurements

In this study, 46 patients with TN (31 females and 15 males) and 35 healthy controls (18 females and 17 males) were included. The demographics and clinical characteristics of the TN patient group are listed in Table 1. No significant differences were observed between the two groups in terms of age and sex distribution.

**Table 1:** Clinical and demographic characteristics.

Items	HC	TN	*p*-value
Gender (male/female)	17/18	15/31	0.145
Age (years)	54.54 ± 7.96	57.69 ± 9.93	0.113
MPQ	–	17.20 ± 3.84	–
FPS	–	79.11 ± 29.98	–
PASS	–	44.26 ± 17.11	–
PCS	–	27.98 ± 11.42	–
Pain duration (years)	–	4.19 ± 5.03	–

HC, healthy controls; TN, patients with trigeminal neuralgia; MPQ, McGill Pain Questionnaire; FPS, Penn Faces Pain Scale; PASS, Pain Anxiety Symptom Scale; PCS, Pain Catastrophic Scale. Data are presented as mean ± s.d.

### 3.2 The results of voxel-based morphometry analysis

The VBM analysis did not find any statistically significant differences in the gray matter volume between patients with TN and HCs (FWE corrected, *p* > 0.05) (Table 2).

**Table 2:** Brain saliences for white-matter fiber tracts in the PLSC analysis.

White matter fiber tracts	Brain salience	*p*-value
*Genu of corpus callosum*		
L	−5.27	1.0479e-08*
R	−5.27	1.0479e-08*
*Middle cerebellar peduncle*		
L	−4.24	5.3520e-06*
R	−4.24	5.3520e-06*
*Splenium of corpus callosum*		
L	−4.22	5.9607e-06*
R	−4.22	5.9607e-06*
*Cerebral peduncle*		
L	−3.75	6.4711e-05*
R	−1.59	0.1056
*Tapetum of the corpus callosum*		
L	–	–
R	−2.44	0.0114
*Posterior thalamic radiation*		
L	−2.24	0.0207
R	–	–
*Posterior limb of internal capsule*		
L	−2.23	0.0213
R	–	–
*Anterior limb of internal capsule*		
L	−0.75	0.4514
R	−1.81	0.0642

*p < 0.01.

### 3.3 The results of regional white matter diffusion property analysis

Compared to the HCs, the TN group exhibited significantly lower FA values in several regions, including the genus of the corpus callosum, bilateral middle cerebellar peduncle, splenium of the corpus callosum, bilateral cerebral peduncle, tapetum of the corpus callosum, left posterior thalamic radiation, left posterior limb of internal capsule, and bilateral anterior limb of the internal capsule in the warm brain regions (*p* < 0.05, FWE corrected) (Figure 1B).

### 3.4 The results of PLS correlation analysis among abnormal white matter, pain intensity, and negative emotion

Multivariate PLS correlation analysis was employed to examine the association of pain intensity, negative emotion, and white matter integrity in patients with TN. The analysis identified four LVs, with only demonstrating significance through the 5,000-permutation bootstrap test: LV1 (*p* = 0.025), LV2 (*p* = 0.851), LV3 (*p* = 0.955), and LV4 (*p* = 0.995). LV1 to LV4 accounted for 98.48, 1.17, 0.24, and 0.12% of the crossblock covariance, respectively. For the LV1, a positive correlation emerged between behavioral scores and brain scores (*r* = 0.366, *p* = 0.014) (Figure 1C). The brain scores are a measure of the similarity between individual brain data and salient brain patterns. Large absolute values of the brain scores demonstrate a strong contribution to the pattern. The mean behavioral correlation coefficient values were averaged from correlation analyzes conducted separately for each behavioral variable using brain scores from the LV1 model: MPQ was 0.35 ± 0.01, FPS was 0.42 ± 0.02, PASS was 0.30 ± 0.01, and PCS was 0.32 ± 0.01 (Figure 1D). The TN group exhibited significantly correlation with pain intensity and negative emotion in the genus of the corpus callosum, bilateral middle cerebellar peduncle, splenium of the corpus callosum, left cerebral peduncle (*p* < 0.01) (Figure 1E; Table 2). The FA value of the genus of the corpus callosum, which area was the most correlation brain region in the PLS correlation analysis, showed a negative correlated with pain intensity and negative emotion (Figure 1F).

**Figure 2:**
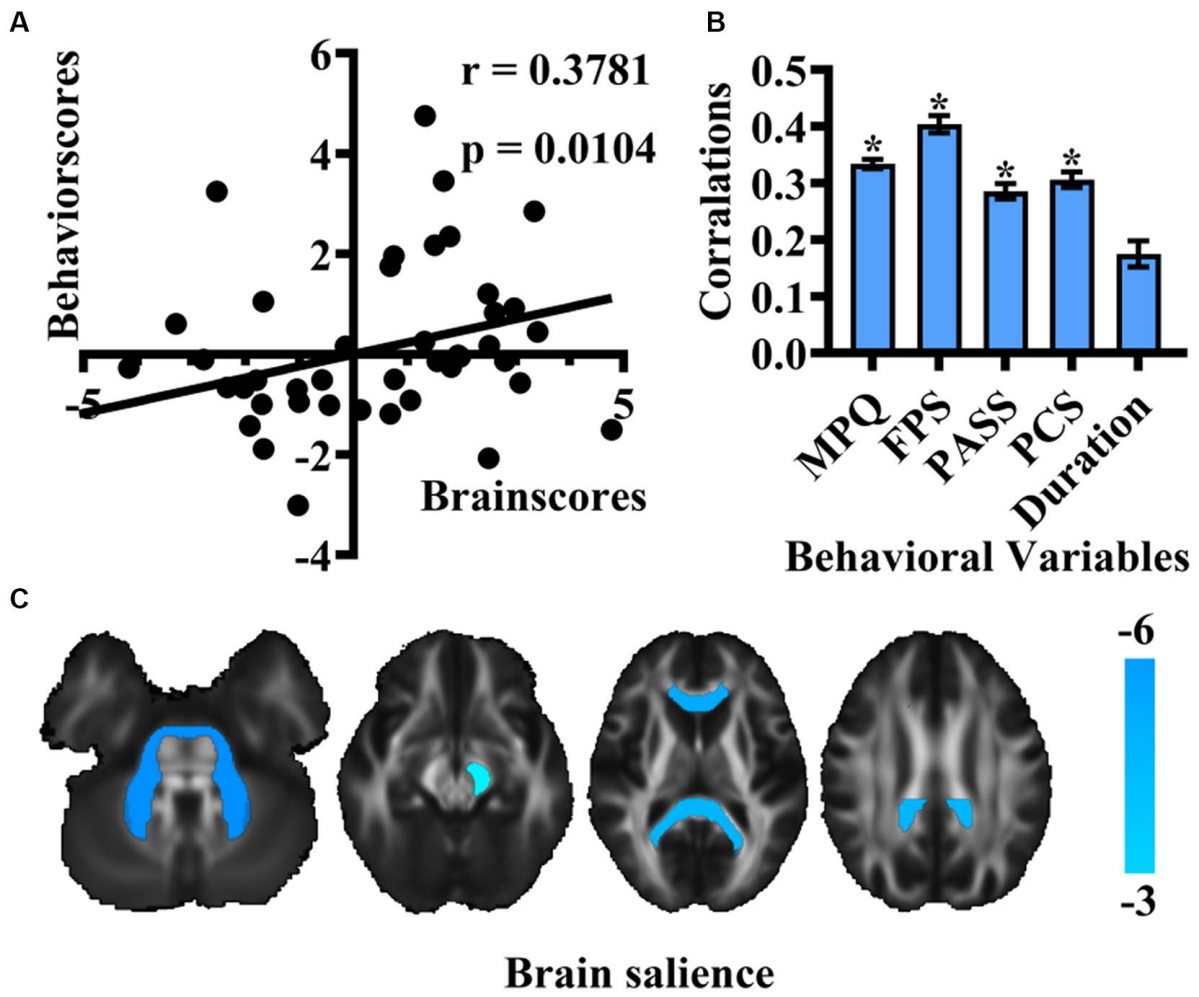
Results of PLS correlation analysis in the association of pain intensity, negative emotion, and pain duration with white matter integrity of patients with TN. **(A)** Scatter plot depicting the correlation between new behavioral scores and brain scores of LV1. **(B)** Bar graph representing Pearson’s correlation coefficients for behavioral variables and brain scores in TN. (* means that the behavioral variable has correlated with brain scores) **(C)** Brain structures exhibiting associations with behavioral variables are highlighted in cold colors. (MPQ, McGill Pain Questionnaire; FPS, Penn Faces Pain Scale; PASS, Pain Anxiety Symptom Scale; PCS, Pain Catastrophic Scale).

Additionally, we incorporated pain duration into behavioral variables to examine the association of pain intensity, negative emotion, and pain duration with white matter integrity of patients with TN. The analysis identified 5 LVs, with only one demonstrating significance through the 5,000-permutation bootstrap test: LV1 (*p* = 0.025), LV2 (*p* = 0.915), LV3 (*p* = 0.871), LV4 (*p* = 0.95), and LV5 (*p* = 1). LV1 to LV5 accounted for 97.53, 1.44, 0.79, 0.2, 0.03% of the crossblock covariance, respectively. This finding indicated that the corresponding brain voxels were significantly associated with the combined effects of the pain intensity, negative emotion and pain duration. A positive correlation was observed between behavioral scores and brain scores (*r* = 0.3781, *p* = 0.0104) (Figure 2A). The mean correlation coefficient values of behavioral variables: MPQ was 0.33 ± 0.01, FPS was 0.40 ± 0.02, PASS was 0.29 ± 0.01, PCS was 0.31 ± 0.01, and for pain duration, it was 0.17 ± 0.02 (Figure 2B). The TN group exhibited significantly correlation with behavioral scores in the genus of the corpus callosum, bilateral middle cerebellar peduncle, splenium of the corpus callosum, left cerebral peduncle (*p* < 0.01) (Figure 2C).

## 4 Discussion

In this study, PLS correlation analysis was employed to investigate the abnormal brain structure associated with pain intensity and negative emotion in patients with TN. The following key results were observed: (1) Significant difference in white matter integrity were identified in patients with TN compared to the HC group; (2) Among the regions displaying abnormal white matter, the most correlation area was the genu of the corpus callosum. Notably, this region exhibited a negative association with both pain intensity and negative emotion. Our findings suggest the involvement of the genu of the corpus callosum in the processing of pain and emotion. In summary, these findings may provide new insights into the neuro-central mechanism of TN.

### 4.1 The integrity decreased of white matter is closely related to TN pain intensity and negative emotion

Pain intensity and negative emotion represent the sensory and affective dimensions of pain, respectively, and are closely linked and interact with each other (Talbot et al., 2019; Gilam et al., 2020; Fan et al., 2021). Due to individual differences in pain intensity and negative emotion in patients with TN, the study of brain structure allows for a more direct correlation of changes with pain and negative emotion. There is ample evidence supporting the presence of white matter structural abnormalities in patients with TN (Zhang et al., 2020; Liu et al., 2022). In our study, we identified white matter regions in the TN group with abnormally lower FA values including the genu of corpus callosum (CC), splenium of CC, tapetum of the CC, bilateral middle cerebellar peduncle (MCP), bilateral cerebral peduncle, left posterior thalamic radiation, bilateral anterior limb of internal capsule, and left posterior limb of internal capsule, compared with the HC group. Low FA value in these regions may indicate compromised health and maturity of the white matter, potentially reflecting maladaptive responses to prolonged, repeated nociceptive input and pain modulation. Some of the abnormal white matter regions in our study align with finding from previous research, such as CC (DeSouza et al., 2014; Hayes et al., 2017; Wang et al., 2017, 2018; Li et al., 2021a,b), corona radiata (CR) (Hayes et al., 2017; Wang et al., 2017, 2018; Liu et al., 2018; Tsai et al., 2018), longitudinal fasciculus (LF) (Tian et al., 2016; Hayes et al., 2017; Liu et al., 2018; Wang et al., 2018), and bilateral internal capsule (Liu et al., 2018). However, the specific physiological changes contributing to the observed low FA in these abnormal white matter regions remain unclear. Two studies (Liu et al., 2018, 2021) have found that low FA values in the anterior CR, external capsule, cerebral peduncle, and genu of CC were significantly negatively associated with pain intensity in TN patients, indicating that the integrity of abnormal white matter regions will decreases as pain worsen. In our study, we observed abnormally low FA values in the TN group (the genu of CC, bilateral middle cerebellar peduncle, splenium of CC, left cerebral peduncle) that were not only associated with high pain intensity but also with high negative emotion. This result suggests that the decreases integrity of neurofibrotic structures in these abnormal white matter regions becomes more significant with increasing pain intensity and negative emotion. A possible explanation is that these white matter regions are common involved in the modulation of the emotional and motivational aspects of pain. The CC, for instance, is formed by neurofibers linking the left and right cerebral hemispheres (Li et al., 2011). Intimately connected to the limbic lobe and belonging to the limbic system, which is involved in behavioral and emotional responses (Atlas et al., 1986). The MCP connect the cerebellum to the pons, serving as the main information afferent pathway to the cerebellum, coordinating and planning motor tasks (Jiang et al., 2020). Diffusion alterations in the cerebral peduncle have also been correlated with changes in motor tasks, sensory, and affective changed (Du et al., 2018), while the posterior thalamic radiation is involved in body perception (Merabet et al., 1998). The internal capsule carries major motor and sensory pathways, transmitting motor, cognitive, visual and sensory information (Cowan and de Vries, 2005). Abnormal diffusion in these tracts may lead to alterations in information integration. Over a prolonged period of pain, these alteration may develop into maladaptive white matter structure, eventually resulting in emotional disorders. Therefore, we hypothesize that the decreased integrity of white matter leads to impaired processing of pain-related emotional and motivational aspects, contributing to negative emotion in patients with TN.

### 4.2 The genu of corpus callosum may be an important region involved in processing pain and emotion

As commonly known, the CC is comprised of fibers connecting both cerebral hemispheres (Li et al., 2011). Anatomically, the CC is divided into distinct regions, including the rostrum, genu, body and splenium (Xue et al., 2018). To date, there is little evidence regarding CC pathology in patients with TN. Two brain imaging studies aimed at exploring the substructure regions’ integrity of the CC in patients with TN found particular damage in the genu (Li et al., 202*1a*) and body (Li et al., 202*1a*,*b*), indicating that as pain intensity progressed, the integrity of various parts of CC decreases to varying degrees. Several studies have highlighted the association between integrity of CC and emotion regulation. The genu and anterior of CC connect the lateral and medial surfaces of the frontal lobes that are thought to be relevant to mood regulation (Standring, 2005). Lischke et al. (2017) showed a correlation between borderline personality disorder individuals’ suicidal behavior and low FA in the splenium and the genu of CC; Gan et al. (2016) found that borderline personality disorder individuals have a low FA in the body and the genu of CC. Matsuo et al. (2010) found that the genu of CC predicted higher impulsivity among suicidal bipolar disorder patients. Additionally, Li et al. (2011), in a study investigating microstructural abnormalities of the CC in adult patients with migraine without aura complicated by depressive/anxious disorders, reported low FA in the entire CC in migraine patients with depressive/anxious disorders, negatively correlation with anxiety and depression scores. In our current study, we further employed PLS correlation analysis and identified the genus of CC as the most correlation white matter regions in patients with TN, showing a negative correlation with both pain intensity and, negative emotion. This suggests that with the deteriorating progression of the genu of the CC, pain intensity and negative emotion become more pronounced. It is noteworthy that the low integrity of white matter regions in patients with TN is localized to the genu of the CC, aligning with findings from previous studies. This indicates that the genu of the CC is particularly vulnerable in TN. The diminished integrity of the genu of the CC may serve as a structural marker indicating disturbed inter-hemispheric structural connectivity. Considering the CC’s role in integrating and transferring emotional, cognitive, motor, and sensory information, the low integrity of the CC in patients with TN suggests a deficit in the ability to control or regulate pain and emotion. Consequently, we conclude that the compromised integrity of the genu of the CC may serves as a neuroanatomical basis for patients with TN complicated by negative emotion.

### 4.3 Pain duration may not be a significant influencing factor for changes in brain structure in patients with TN

Neuroimaging studies have aimed to explore the correlation between alterations in brain structure and pain duration in patients with TN through univariate analysis. These studies have consistently demonstrated that decreased gray matter volumes and disruption of white matter integrity in patients with TN are negatively associated with pain duration (Obermann et al., 2013; Tian et al., 2016; Li et al., 2017, 2018; Tsai et al., 2018). This suggests that longer duration of TN contributes to the observed impairment in brain structure. In our study, we augmented clinical behavioral scores with pain duration to investigate correlation between multivariate behavioral scores and abnormal white matter. Unfortunately, the correlation between pain duration and brain score was not significant in our study. Our findings suggest that pain intensity and negative emotion are crucial influencing factors for abnormal white matter regions in patients with TN, whereas pain duration may not play a significant role. It is essential to note that this result may be attributed to the relatively small sample size.

### 4.4 Gray matter volume as a limited indicator of change in patients with TN

Our research findings indicated that the overall gray matter volume in patients with TN did not exhibit a significant difference compared to HCs. Pain, being a multi-dimensional composite sensation, involves a complex neural network. The structural changes in the brain may be intricately linked with the pain perception and modulation. Zhang et al. (2018), in these exploration of structural and functional deficits in pain-related and emotion-related networks in TN patients, reported that pain relief had protective effects on brain functional connectivity. Existing evidence supports the idea that regional gray matter decreases can partially return to normal following successful amelioration of chronic pain (Gwilym et al., 2010; Moayedi et al., 2011; Čeko et al., 2015). One potential explanation for our results could be that the subjects included in our study experienced pain relief due to medication, resulting in minimal changes in gray matter volume. Additionally, VBM analysis, being a voxel-level analysis methods, provides volume estimations of gray matter (Garrido et al., 2009), but it may neglect potential changes caused by complex folding conditions, including cortical thickness and sulcal depth. Ge et al. (2023) reported a decrease in cortical thickness in the left post central regions of patients with TN compared to HCs, along with a decrease in gray matter volume in the right temporal lobe and right precentral region. The differing processing procedures of various methods can yield distinct results. Another plausible explanation for our finding is that the alteration in the gray matter microstructure in our subjects might not be substantial enough to induce significant changes in gray matter volume.

## 5 Limitations and future directions

Our study is subject to several limitations that should be considered. First, the correlation between disease duration and brain score was not significant in our study may be influenced by the small sample size of the patient group. Second, while pain comprises seven dimensions, our study included two dimensions (pain intensity and negative emotion), potentially limiting the comprehensive understanding of pain in patients with TN. Third, majority of the patients in our study were using pain-relief medications before image collection, which might influence the brain structural changes. Finally, our study is a cross-sectional in nature and lacks a longitudinal imaging data post-surgical treatment. Future studies should aim to increase sample sizes, incorporate more dimensions of pain-related indicators, mitigate the influence of drugs, and design longitudinal analyses to better understand the dynamic changes over time.

## 6 Conclusion

Innovatively employing partial least squares correlation analysis, our study explored the relationship among pain intensity, negative emotion and white matter microstructure in patients with TN. The result revealed that the genu of the corpus callosum as the most correlation brain region, exhibiting a negative association with both pain intensity and negative emotion. Indicated that the genu of corpus callosum plays an important role in the cognition of pain perception, the generation and conduction of negative emotions in patients with TN. These findings contribute novel evidence to the understanding of abnormalities in the genu of corpus callosum and its role in the pathophysiology of TN.

## Data availability statement

The original contributions presented in the study are included in the article/supplementary material, further inquiries can be directed to the corresponding author.

## Ethics statement

The studies involving humans were approved by the Ethics Board of the Affiliated Hospital of North Sichuan Medical College. The studies were conducted in accordance with the local legislation and institutional requirements. The participants provided their written informed consent to participate in this study.

## Author contributions

BS: Writing – original draft. CZ: Writing – review & editing, Funding acquisition. KH: Software, Writing – original draft. AB: Writing–review & editing. XY: Investigation, Writing – original draft. CJ: Investigation, Writing – original draft. HLi: Methodology, Writing – original draft. HLu: Resources, Writing – original draft. QZ: Data curation, Writing – original draft. HY: Writing–review & editing.

## Footnotes

^1^http://www.fmrib.ox.ac.uk/fsl

^2^http://www.fmrib.ox.ac.uk/fsl/data/FMRIB58_FA.html

^3^http://www.rotman-baycrest.on.ca/pls/runningon
